# Reporting Quality of Randomized Controlled Trials in Antineutrophil Cytoplasmic Autoantibody–Associated Vasculitis

**DOI:** 10.1016/j.ekir.2025.07.012

**Published:** 2025-07-16

**Authors:** Vanja Ivković, Annette Bruchfeld, Lauren Floyd, Sarah Soyeon Oh, Peong Gang Park, Jae Il Shin, Andreas Kronbichler

**Affiliations:** 1 Department of Health, Medicine and Caring Sciences, Linköping University, Linköping, Sweden; 2 Department of Renal Medicine, Karolinska University Hospital and CLINTEC Karolinska Institute, Stockholm, Sweden; 3 Department of Renal Medicine, Royal Preston Hospital, Lancashire Teaching Hospitals NHS Foundation Trust, UK; 4 Institute of Global Engagement & Empowerment, Yonsei University, Seoul, Republic of Korea; 5 Department of Pediatrics, Ajou University School of Medicine, Suwon, Republic of Korea; 6 Division of Pediatric Nephrology, Department of Pediatrics, Severance Children’s Hospital, Yonsei University College of Medicine, Seoul, Republic of Korea; 7 Institute of Kidney Disease Research, Yonsei University College of Medicine, Seoul, Republic of Korea; 8 Department of Internal Medicine IV, Nephrology and Hypertension, Medical University Innsbruck, Innsbruck, Austria

**Keywords:** ANCA-associated vasculitis, randomized controlled trials, reporting quality

## Abstract

**Introduction:**

Treatment of anti-neutrophil cytoplasmic antibody–associated vasculitis (AAV) relies on high-quality randomized controlled trials (RCTs). Comprehensive and transparent reporting is crucial for the integrity and interpretation of trial findings. Consolidated Standards of Reporting Trials (CONSORT) checklists are part of a global initiative aiming to improve the quality of RCTs through standardized reporting. Reporting quality of AAV trials is unknown, thus we aimed to evaluate their adherence to CONSORT.

**Methods:**

A PubMed search for RCTs in AAV resulted in 596 hits. These were filtered to include only original RCTs published between January 2011 to March 2025. Adherence was calculated as the total percentage of all items applicable to the trial.

**Results:**

A total of 31 RCTs enrolling 3421 patients were included. Only 3 trials (10%) mentioned adherence to the CONSORT 2010 statement checklist, with only 1 including it as supplementary material. RCTs adhered to a median of 83% (interquartile range [IQR]: 77%–86%) CONSORT 2010 statement checklist items. Adherence was moderate (50%–75%) for 5 items and low (< 50%) for 6 items. There was no difference in adherence between industry-sponsored and other trials. Adherence was lowest from 2011 to 2013 (69%) but increased and remained between 79% and 86% afterward. RCTs had a mean adherence to CONSORT-Abstracts (CONSORT-A) of 73% ± 15% (min–max: 41%–94%).

**Conclusion:**

Although the overall adherence of RCTs in AAV to both CONSORT checklists is good, the reporting of some key items, such as randomization, blinding, adherence to trial protocol, and similarity of interventions is lacking.

## Graphical abstract

**Figure:**
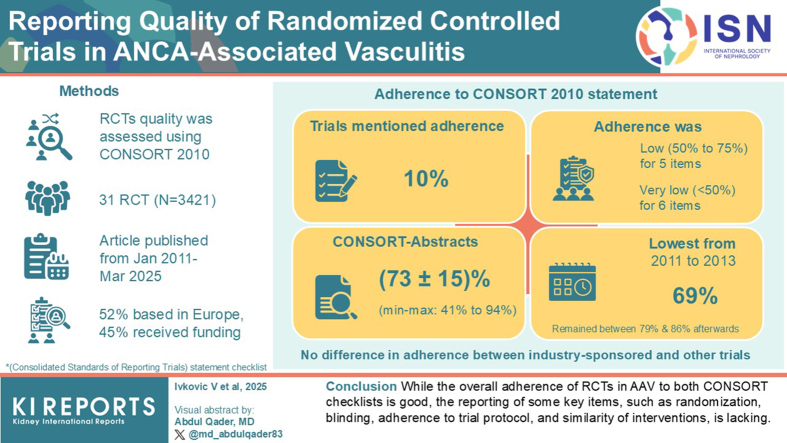

ANCA-associated vasculitis (AAV) is a multisystem immune-mediated disease frequently affecting the kidneys and the respiratory tract.^1^^,^^2^ Treatment of AAV is reliant on evidence generated from RCTs, some of which have changed clinical care practices, especially in the past 15 years.^3^, ^4^, ^5^, ^6^, ^7^, ^8^ Well-designed RCTs are considered the pinnacle of research design, providing the highest-grade evidence in medicine.^9^ Comprehensive and transparent reporting of RCTs is crucial for the integrity and interpretation of their findings, obtaining valid and high-quality meta-analyses, and translation of evidence to clinical practice. Enhancing the Quality and Transparency of Health Research (EQUATOR) was a global initiative aiming to standardize reporting and improve the quality of RCTs. Enhancing the quality and transparency of health research devised a set of widely-used statements and checklists, which included a broad array of items relating to different facets of trial design and presentation.^10^ These included their most prominent tool, CONsolidated Standards Of Reporting Trials (CONSORT) 2010, a checklist of 37 items assessing the reporting of trial design and methodology, for example, randomization, allocation concealment, blinding, as well as of trial outcomes.^11^^,^^12^ This was followed by a set of extensions which were tailored to assess specific parts of trials or different trial types, such as CONSORT for Abstracts (CONSORT-A), which assesses the reporting of trial abstracts and structured summaries.^13^

An uptick in RCTs performed in the field of nephrology was observed in the past decade; however, some studies have shown that they may lack clarity on design and methodology.^14^, ^15^, ^16^, ^17^, ^18^, ^19^, ^20^ There is currently no evidence on the quality of reporting in AAV RCTs. In this systematic review, we aimed to provide data on the reporting quality of AAV trials, analyzing their adherence to CONSORT 2010 checklist as well as adherence of trial abstracts to CONSORT-A checklist, temporal trends, and factors associated with adherence.

## Methods

### Search Strategy and Selection of Studies

We searched PubMed for RCTs published from January 1, 2011 to March 7, 2025 on granulomatosis with polyangiitis, microscopic polyangiitis, and eosinophilic granulomatosis with polyangiitis. No filters or limiters were applied. The following search string was used:

(("Anti-Neutrophil Cytoplasmic Antibody-Associated Vasculitis"[Mesh]) OR ("ANCA associated vasculiti∗" [tiab]) OR ("churg-strauss" [tiab]) OR ("wegener" [tiab]) OR ((("granulomatosis"[tiab]) OR ("microscopic"[tiab])) AND ("polyangiitis"[tiab])) OR ((("ANCA"[tiab]) OR ("Antibodies, Antineutrophil Cytoplasmic"[Mesh]) OR ("antineutrophil cytoplasmic antibod∗"[tiab])) AND (("vasculitis"[tiab]) OR ("glomerulonephritis"[tiab]) OR ("renal"[tiab])))) (randomized or randomization or randomised or randomisation).

**Figure 1:**
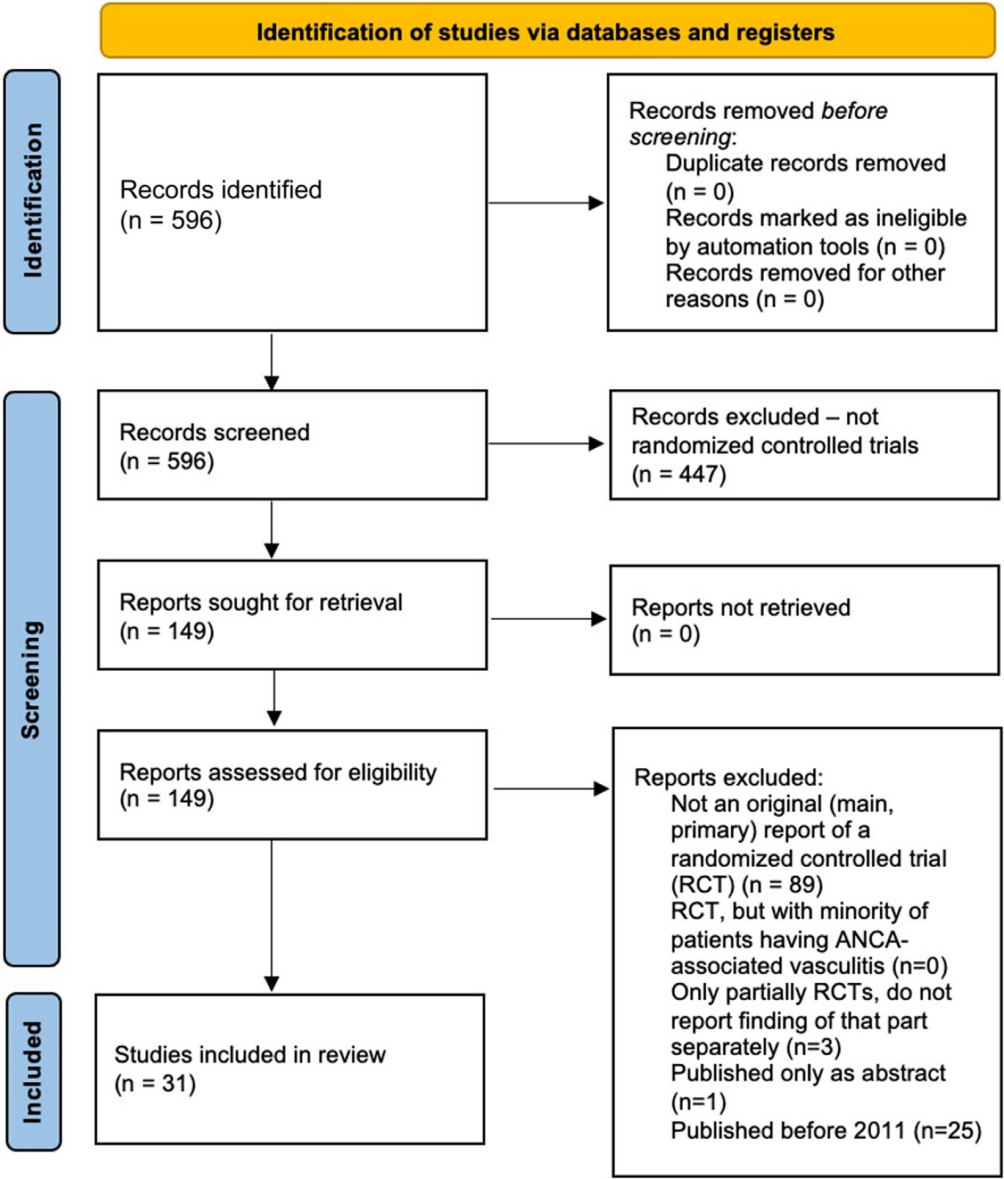
PRISMA diagram of included studies.

The initial search resulted in 596 hits, which were further filtered through 3 steps to include only original (main) reports of RCTs. In the first step, we inspected abstracts and, where needed, full texts to exclude all studies which were not RCTs (*n* = 447). Out of the remaining 149 studies, we excluded studies according to these prespecified criteria (as defined when registering study in PROSPERO): (i) studies which were not main reports of RCTs (*n* = 89), (ii) studies which enrolled only a minority of patients with AAV (*n* = 0), (iii) studies where only a part of the study is an RCT and no separate data on the results of this part are provided (*n* = 3), and (iv) studies that were published only as an abstract and not as full text article (*n* = 1). In the last step, we excluded all trials published before 2011 (*n* = 25) (Figure 1).

### Data Extraction

Data extraction was performed using a predefined sheet to systematically extract the data on all included RCTs. Author, VI extracted all data and entered it into the data extraction sheet. A second author (LF) extracted and verified grading for a random sample of 20% of included RCTs. The absolute agreement between the 2 authors was > 75% for all comparisons, specifically 0.76 and 0.83 for CONSORT 2010 score and percentage, respectively; and 0.94 and 0.92 for CONSORT-A score and percentage, respectively. All questions were discussed between the 2 authors and then intended to be discussed with the whole review team, if needed (this last step was not needed because the 2 authors reached a consensus on all disagreements).

### Assessment of Reporting Quality

Reporting quality of RCTs was assessed using the CONSORT 2010 statement checklist which includes 37 items (Supplementary Material). Trial abstracts were checked against the CONSORT-A statement checklist which consists of 17 items (Supplementary Material). An adjustment or applicability period of 1 year after statement publication was provided; thus, all trials published from 2011 were checked for concordance with CONSORT (published in 2010) and their abstract for concordance with CONSORT-A.

For both statement checklists, we provided 1 point if the item was adequately reported in the trial and no points if it was not. We did not provide any fractional points for partially adequate reporting. If the item was not applicable for that trial, for example, for 11a “If done, who was blinded after assignment to interventions (for example, participants, care providers, those assessing outcomes) and how” for all open-label or unblinded trials, it was skipped and the maximum score for that trial was reduced by 1 point, which was done to remove penalization for nonapplicable requirements. Finally, to account for “not applicable” items, which was not apparent when expressing adherence as an absolute score, we calculated the percentage of adherence with both statement checklists for each trial and then obtained a summary assessment for all trials.

### Statistical Analysis

We used Kolmogorov-Smirnov test to assess normality of distribution of continuous variables. These were presented variables as mean and SD (normally distributed variables) and median and IQR (nonnormally distributed variables). We presented categorical variables as absolute value and percentage. Between-group comparisons were compared using *t* test (normally distributed variables) or Mann-Whitney U-test (nonnormally distributed variables). Analysis of variance and Kruskal-Wallis tests were used for comparison of normally and nonnormally distributed variables, respectively, with 3 or more groups. Fisher exact test was used to test differences in distribution of categorical variables. Correlations were tested using Pearson’s test (normally distributed variables) or Spearman’s rank correlation test (nonnormally distributed variables). Two-tailed *P*-value < 0.05 was used as cut-off for statistical significance.

### Ethical Statement, Compliance With Relevant Statements, and Registration

Ethical approval was not needed because the review only collected existing data provided in research papers. This review is compliant with Preferred Reporting Items for Systematic Reviews and Meta-Analyses, and the relevant checklist has been added to Supplementary Material. The review was registered in PROSPERO (CRD420250649555).

## Results

### Characteristics of Included RCTs

The search resulted in a total of 596 hits of which 31 trials were published from January 2011 to March 2025 and were included (Table 1) (see Supplementary Table S1 for details). The trials included a total of 3421 patients (median: 95, IQR: 42–129 patients; 47% women). Of the 21 trials which reported trial phase, 4 were phase 2, 11 were phase 3, and 6 were phase 4. A total of 25 trials (81%) were superiority trials with a total of 23 (74%) being multicenter. Ten (32%) were blinded and 18 trials (58%) had adequate allocation concealment. Of the included trials, 26 trials (84%) evaluated a pharmacologic intervention with 8 having placebo as comparator. With respect to geographic distribution, 16 trials (52%) were based in Europe, 6 (19%) in Australasia, 2 (6%) in the Americas, and 7 (23%) trials were global (defined as conducted in 2 or more continents). In total, 14 trials (45%) received industry funding.

**Table 1:** Characteristics of the included randomized controlled trials (*n* = 31), including key aspects such as clinical phenotypes of patients and relevant measures of adherence

Characteristic	Value (%)
Enrolment	
Number of patients	3421
Women	1606 (47)
Clinical phenotype	
GPA	22 (79)
MPA	23 (82)
EGPA	8 (29)
Not specified	2 (6)
Number of centers	
Single center	8 (26)
Multicenter	23 (74)
Trial type	
Superiority	25 (81)
Non-inferiority	6 (19)
Blinding	
Double	10 (32)
Single	0 (0)
None	21 (68)
Allocation concealment	
Adequate	18 (58)
Intervention	
Pharmacological	27 (87)
Placebo-controlled	8 (26)
Analysis	
ITT or modified ITT	25 (81)
Other	6 (19)
Outcome adjustment	
Adjusted	1 (3)
Non-adjusted	30 (97)
Composite	5 (16)
Events	
Number of events	1359
Time-to-event outcome	28 (90)
Other outcomes	3 (10)
Funding	
Industry-supported	7 (23)
Government-supported	16 (51)
Industry + governmental support	7 (23)
None	1 (3)
Region	
Europe	16 (52)
Australasia	6 (19)
Americas	2 (6)
Global	7 (23)

EGPA, eosinophilic granulomatosis with polyangiitis; GPA, granulomatosis with polyangiitis; ITT, intention-to-treat; MPA, microscopic polyangiitis.

### Adherence to CONSORT 2010 Statement Checklist

A total of 29 trials (94%) were registered in clinical trial databases, 76% of them in ClinicalTrials.gov. Aside from including CONSORT flowchart diagram, only 3 trials (10%) mentioned adherence to CONSORT statement checklist and only 1 of these included it as a supplementary material. RCTs had a median of 28 (IQR: 26–30) points and adhered to a median of 83% (IQR: 77%–86%, min–max: 61%–94%) items. Fifteen of 20 journals (75%), which published the trials included the need for adherence to CONSORT 2010 in their submission guidelines, and 8 journals (40%) asked that the list be uploaded or made available at editors’ request.

Adherence to specific CONSORT 2010 items was grouped into the following: (i) low adherence (< 50%), (ii) moderate adherence (50%–75%), and (iii) high adherence (> 75%). It was highest, ranging from 75% to 100%, across several key domains. These included providing structured trial summary (1b) (100%); providing scientific background, rationale, and objectives (2a and 2b) (both 100%); defining eligibility criteria for trial participants (4a) (100%); and reporting interventions for each trial arm (5) (100%). High adherence was observed in defining prespecified main outcomes (6a) (97%), providing details on methods used to generate the random allocation sequence (8a) (84%) and reporting statistical methods and methods for subgroup or adjusted analyses (12a and 12b) (100% and 96%, respectively). Trials frequently reported the number of participants randomized to each arm (13a) (100%), number of patients lost to follow-up (13b) (100%), dates of recruitment and follow-up (14a) (97%), or reason the trial was stopped early (14b) (100%). In addition, all studies included a table with baseline characteristics (15) (100%), provided the number of participants in analysis (16) (100%), the effect size estimates for the main outcomes (17a) (100%) as well as the absolute and relative effect sizes for binary outcomes (17b)(100%). Most studies provided results of subgroup and adjusted analyses (18) (96%), discussed all relevant harms or unintended effects (19) (94%), limitations (20) (90%), generalizability of findings (21) (94%), providing an interpretation consistent with results (22) (100%), giving registration number (23) (94%), location where the full protocol can be accessed (24) (90%), and information on the source of funding and other support (25) (90%).

**Figure 2:**
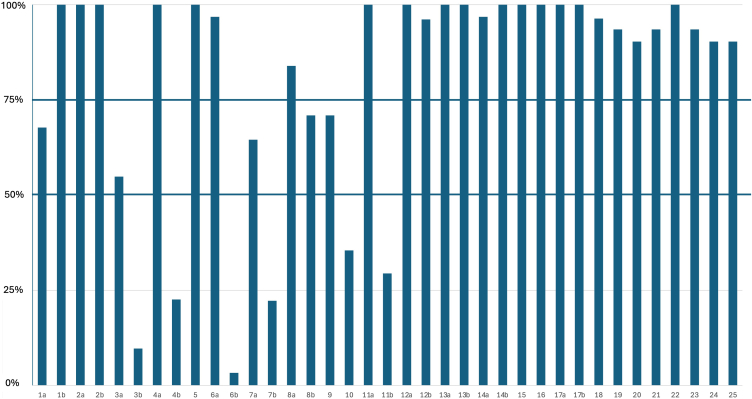
Adherence to specific CONSORT 2010 statement checklist items. The following 5 items had moderate adherence (50%–75%): identification as a randomized trial in the title (1a), description of trial design (3a), method on how sample size was determined (7a), type of randomization (8b), and allocation concealment (9). The following 6 items had low adherence (< 50%): reporting whether there were important changes to methods reflected in protocols amendments after trial commencement (3b), clear description of settings and locations of the trial (4a), reporting if any changes to trial outcomes have been made after trial commencement (6b), explanation of interim analyses and stopping guidelines (7b), noting who generated the random allocation sequence (10), and mentioning if there was similarity of interventions (11b). The full CONSORT 2010 checklist is presented in the Supplementary Material. CONSORT, Consolidated Standards of Reporting Trials.

All studies using blinding described who was blinded to assignment of interventions and all 4 trials which were prematurely terminated have noted that in the main text. Items which had lower adherence (50%–75%) were the following: identification as a randomized trial in the title (1a) (68%), description of trial design (3a) (55%), method on how sample size was determined (7a) (65%), type of randomization (8b) (71%), and allocation concealment (9) (71%). Lowest adherence (< 50%) was for the following: reporting whether there were important changes to methods reflected in protocols amendments after trial commencement (3b) (10%), clear description of settings and locations of the trial (4b) (23%), reporting if any changes to trial outcomes have been made after trial commencement (6b) (3%), explanation of interim analyses and stopping guidelines (7b) (22%), noting who generated the random allocation sequence (10) (35%), and mentioning if there was similarity of interventions (11b) (29%). Adherence to specific CONSORT 2010 statement checklist items is shown in Figure 2.

**Figure 3:**
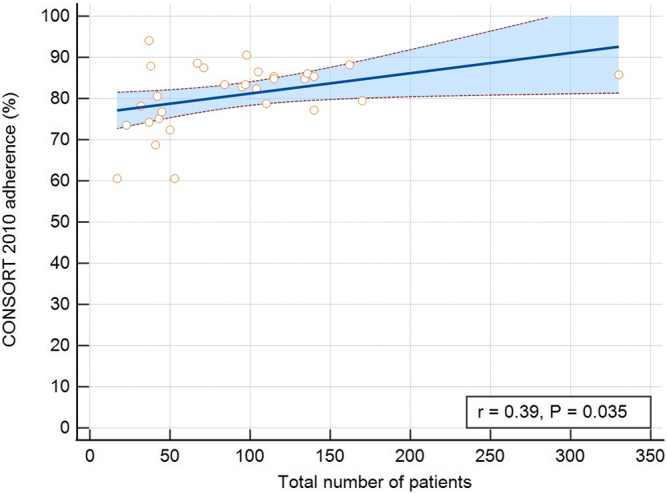
Association of adherence to CONSORT 2010 statement checklist and trial size. Adherence was positively correlated with the number of patients enrolled in the trial. CONSORT, Consolidated Standards of Reporting Trials.

**Figure 4:**
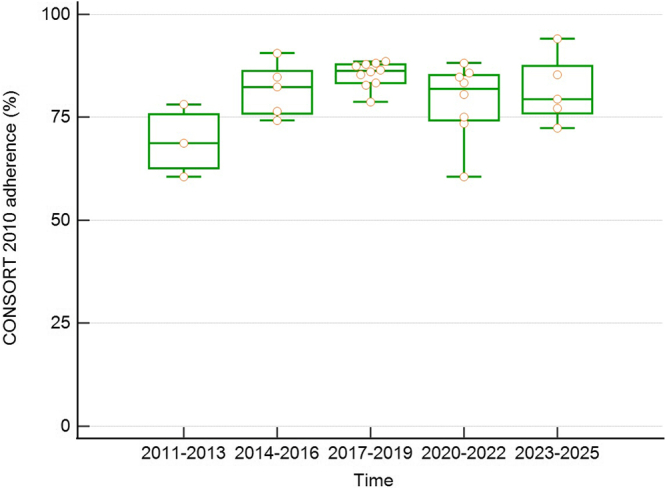
Adherence to CONSORT 2010 statement checklist and publication period. Adherence was low in the oldest studies (published from 2011 to 2013) but increased in subsequent time periods. CONSORT, Consolidated Standards of Reporting Trials.

The percentage of adherence did not show temporal dynamics (*r* = 0.04, *P* = 0.83) but was correlated with RCT size (*r* = 0.39, *P* = 0.035) (Figure 3). However, when focusing on specific adherence rates in 3-year periods, adherence to CONSORT was lowest in the earliest period (2011–2013) at 69%. In subsequent periods, adherence remained stable between 79% and 86% (*P* for trend = 0.06) (Figure 4).

There was no difference in percentage of adherence to CONSORT statement checklist between industry-sponsored and other trials, continent where the trial was performed, trials of different phase, clinical phenotype of AAV, trial type, blinding, intervention type, placebo-control, or type of analysis (Table 2).

**Table 2:** Comparison of adherence to CONSORT 2010 statement checklist across randomized controlled trial characteristics

Characteristic	CONSORT 2010 (%)
Clinical phenotype	
GPA	83
MPA	83
EGPA	80
Number of centers	
Single-center	77
Multicenter	83
Trial type	
Superiority	82
Noninferiority or equivalence	86
Intervention type	
Pharmacologic	83
Nonpharmacologic	77
Blinding	
Yes	85
No	82
Placebo-controlled	
Yes	85
No	82
Analysis	
ITT/modified ITT	83
Other	78

CONSORT, Consolidated Standards of Reporting Trials; EGPA, eosinophilic granulomatosis with polyangiitis; ITT, intention-to-treat; GPA, granulomatosis with polyangiitis; MPA, microscopic polyangiitis.

### Adherence to CONSORT-Abstracts Statement Checklist

**Figure 5:**
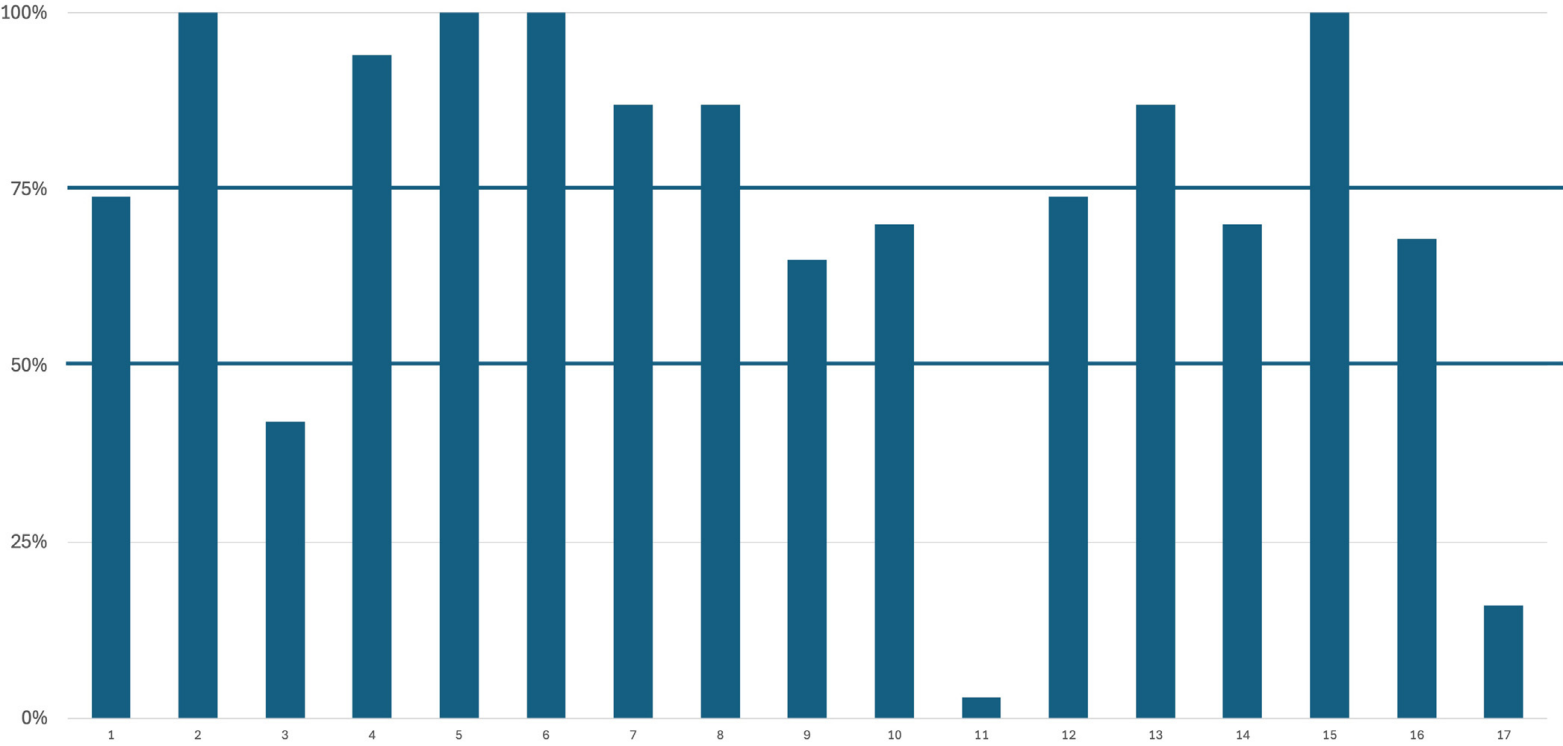
Adherence to specific CONSORT-Abstract statement checklist items. Adherence was lowest (< 50%) for: description of the trial design (3), providing trial status (11) and providing information on source of funding (17). The full CONSORT Abstracts checklist is presented in the Supplementary Material. CONSORT, Consolidated Standards of Reporting Trials.

The trials had a mean score of 12.3 ± 2.6 (out of maximum of 17) and adhered to a mean of 73% ± 15% (min–max: 41%–94%). Adherence to specific CONSORT-A statement checklist items is shown in Figure 5. Although all trial abstracts reported information on the correspondence author, interventions for each group, objectives, and interpreted the results appropriately, few abstracts described the trial design (42%), provided information on whether blinding was used (65%), provided trial status (3%) and provided information on the source of funding (16%).

There was no difference in the percentage of adherence to CONSORT-A statement checklist between trials of different phase, clinical phenotype of AAV, blinding, intervention type, placebo-control, or type of analysis. Noninferiority and equivalence trials had higher adherence than superiority trials (Table 3). There was some geographical imbalance with highest adherence in global trials (89%) and those performed in Americas (77%) than European (68%), and Australasian (66%, *P* = 0.005) trials.

**Table 3:** Comparison of adherence to CONSORT Abstracts checklist across randomized controlled trial characteristics

Characteristic	CONSORT abstracts (%)
Clinical phenotype	
GPA	77
MPA	76
EGPA	80
Number of centers	
Single center	64^a^
Multicenter	76^a^
Trial type	
Superiority	70^a^
Noninferiority or equivalence	85^a^
Intervention type	
Pharmacologic	74
Nonpharmacologic	64
Blinding	
Yes	81^a^
No	69^a^
Placebo-controlled	
Yes	78
No	71
Analysis	
ITT/modified ITT	74
Other	68

CONSORT, Consolidated Standards of Reporting Trials; EGPA, eosinophilic granulomatosis with polyangiitis; ITT, intention-to-treat; GPA, granulomatosis with polyangiitis; MPA, microscopic polyangiitis.

a Statistically significant comparison.

## Discussion

In this review, we provided evidence on reporting quality of AAV RCTs, specifically, their adherence to CONSORT 2010 statement and CONSORT-A checklists. The trials had a relatively good overall adherence to both checklists, with an adherence to CONSORT 2010 of 83% and to CONSORT-A of 73%. Larger trials have, in general, better adherence to CONSORT 2010. An analysis of the time periods showed that adherence was lower in earlier trials; however, researchers adapted to and accepted CONSORT relatively quickly after the first 4 years after publication, keeping the adherence rates high afterward. Our results are comparable to adherence reported across other fields of medicine.^21^, ^22^, ^23^, ^24^, ^25^, ^26^, ^27^, ^28^, ^29^, ^30^

Although adherence to CONSORT 2010 as a whole was high, there was great variability among items, with rates for several specific items being moderate or low. In total, 5 items had adherence between 50% and 75% and 6 items had adherence < 50%, with some items having ≤ 10%. This is in line with a study encompassing 118 trials in nephrology published in top-ranked journals in 3 nonconsecutive years (1996, 2006, and 2016), which showed that < 2% of studies reported any changes to trial design whereas > 90% reported statistical methods for group comparisons of the primary and secondary outcome.^16^ As for CONSORT-A, the main points which would need addressing in most trial abstracts are reporting of trial status and registration (very few trials reported if the trial is ongoing, recruiting, finished, etc.), reporting of trial funding (it would be useful to report this in the abstract to enable visibility and promote transparency), and description of blinding and of trial design (full description is helpful, but is rarely observed). Items with low adherence are the weak links on which the trial quality hinges and should become the focus early in the trial design phase.

We would like to emphasize 3 areas where particular attention should be given to improve reporting of trials. First, randomization is crucial and should be explained in detail in all trials. Whereas some items describing randomization had relatively good adherence, 29% of trials did not report adequate allocation concealment and only 35% mentioned who generated the random allocation sequence. Strippoli *et al.*^14^ in their review of 430 RCTs in nephrology published between 1966 and 2002 reported that only 7.4% of RCTs used adequate allocation concealment, with little variation over time. A review focusing on a subset of 4 CONSORT items describing randomization found that among 74 included trials in nephrology, only 4 trials (6.8%) reported sufficiently on all 4 items. Moreover, 19 trials (25.7%) provided no information at all on randomization methodology. When focusing on specific items, 40.5% of trials reported on sequence generation, 70.8% reported on randomization type, 41.9% reported on allocation concealment, and 13.5% reported on randomization implementation.^18^ These deficiencies are not merely academic. There is evidence that insufficient reporting of randomization is associated with trials reporting larger effect sizes, which might indicate less reliable results and the potential for bias.^31^ As a result, proper randomization is an important aspect of trial methodology, lack of which undermines the credibility and reproducibility of findings.

Second, out of all trials in which this was feasible, only 29% mentioned if any steps were taken to assure similarity of interventions, for example, identical pills or opaque infusion containers. Although ensuring that interventions are mutually unrecognizable is not always feasible, for example, when evaluating plasma exchange, it is indeed possible to achieve this in most trials. Moreover, aside from low rates of allocation concealment and assuring similarity of interventions, only 32% of trials used blinding. Comparable findings have been reported in the review by Strippoli *et al.*,^14^ in which 43.0% of trials had adequate blinding. All these point to glaring gaps in masking, which should be bridged to improve trial design and achieve reliable outcomes.

Lastly, any changes to the protocol which have indeed been made in several trials, have very seldom been commented on in the paper. Monitoring and reporting changes in trial protocol is important to correctly assess chances of bias. Alterations to primary outcomes, eligibility criteria, statistical analysis plan, and sample size calculations can all have a significant impact on the trial results and interpretation. When discrepancies exist between original protocols and reported outcomes, they should be clearly disclosed and justified within the publication.

Interestingly, though all top-ranked nephrology journals mention the need for adherence to CONSORT statement checklist in their authors’ guidelines, only 3 trials included in our review mentioned adherence to CONSORT and only 1 included the completed checklist in the Supplementary Material. Although CONSORT is indeed ubiquitously mentioned in authors’ guidelines, the strength of the recommendations varies across journals. *Kidney International* and *Kidney International Reports* state that the authors “must adhere” to the CONSORT statement and “must upload” the checklist; *NDT* and *CKJ* mention that the author “must comply” to the CONSORT statement; *AJKD* mentions that the authors “should follow” CONSORT statement, and *JASN* and *CJASN* “encourage authors” to comply with CONSORT statement. None of these journals mention CONSORT-A extension for abstracts of RCTs. The situation in other nephrology journals is worse and suggests that although there is explicit endorsement, there is limited enforcement. A 2024 study surveyed the reporting of guideline and clinical trial registration policies in 62 journals in nephrology. A total of 52% of journals required clinical trial registration. However, only 17.7% of them required adherence to CONSORT.^32^ To improve compliance, both authors and peer reviewers could be required to submit completed CONSORT and CONSORT-A checklists as part of the submission and review process, ensuring that adherence is actively verified rather than passively encouraged. Journals can include specific forms for authors and reviewers to acknowledge the awareness of and adherence to the checklists on multiple levels. Furthermore, awareness of the importance of the standardized checklists and the correct way to assess RCT reporting quality can be included as part of training for new editors and reviewers and brief tests and surveys of the knowledge and grading skills in practice can be performed periodically.

Nevertheless, identifying the most effective strategy to improve reporting in RCTs remains a challenge. A previous study has shown that quality of reporting is better in journals which are “CONSORT promoters,” defined by inclusion of the checklist in the journal’s information to author or a requirement that the authors complete the CONSORT checklist.^11^ However, CONSORT-PR, an RCT developed to evaluate whether asking peer reviewers to check adherence to CONSORT statement checklist will improve reporting, showed that there is no difference in percentage of adequate reporting of 10 selected, important and frequently poorly reported items between reviewers who were specifically asked to check them and those who were not.^33^

There are some limitations to our study. We used the general CONSORT 2010 statement, which is by far the most widely used. This checklist is intended to be used for all types of RCTs and is adequate and suitable to grade any such trial. Subsequent extensions to this CONSORT statement have been devised to provide a more in-depth assessment of certain parts of the study or be used for specific trial types; for example, for assessment of harms, for surrogate end points, for outcomes, for crossover trials, for factorial trials. Although several of these extension statements would be applicable to some of the trials we included and might provide a more closely fit evaluation for these particular trials, such extensions could be applied only for a fraction of the included trials. Nevertheless, even if we included some of the extensions and data which would provide us with more information on specific trials, this could not be used to contribute to the aggregate data or compare to the reporting quality of the other trials which were assessed using the general CONSORT 2010 checklist. Therefore, we did not use any of these extensions, except CONSORT-A, to assess included trials because we wanted to provide a broad canvassing of the reporting quality of AAV trials and in-depth assessment of different design aspects or types of trials was not our aim; however, these aspects are of interest and can be explored in a future study. Furthermore, we used binary (yes/no) scoring for grading checklist adherence providing 1 point for adherence and zero points for no-adherence. Although this scoring is by far the most widely used and is the one intended for CONSORT checklists, we agree that there might be different approaches such as assigning partial score, for example, half a point, when study adheres to 1 or more parts of a specific checklist item. We chose this approach to enable comparison with other studies and avoid any confusion. However, there might be uncertainty if some CONSORT checklist items might be vague or pertain to multiple different aspects and whether they should, in future incarnations, be split into ≥2 items, which is an interesting question for further research.

Although overall, the adherence of RCTs in AAV to CONSORT 2010 and CONSORT-A is good, the reporting and conduct of some key items, such as randomization, blinding, adherence to trial protocol, and similarity of interventions, is seriously lacking. There is much room for improvement in several facets of trial reporting, which will hopefully translate to more reliable trial outcomes.

## Disclosure

AK received unrestricted research grants from CSL Vifor and Otsuka; and consulting fees from Amgen, AstraZeneca, Boehringer Ingelheim, CSL Vifor, Delta4, GlaxoSmithKline, Novartis, NovoNordisk, Otsuka, Roche, Sobi, and Walden Biosciences. AB received consultant and speaker fees from Alexion, AstraZeneca, Bayer, Boehringer-Ingelheim, CSL Vifor, Fresenius, Otsuka, and GSK; received payment for expert testimony from the Swedish National Board of Health and Welfare; and was a data safety monitoring board member for Alexion, Boehringer-Ingelheim, and CSL Vifor, and an associate editor of CJK and leaderships role of IWG of European Renal Association. VI has received a grant as part of the European Renal Association (ERA) Long-term Fellowship. All the other authors declared no competing interests.

## Supplementary Material

Supplementary File (PDF) **Table S1.** Main characteristics of all included trials. List of all randomized controlled trials included in the analysis. CONSORT 2010 checklist. CONSORT-Abstracts checklist.
