# Supplementary material for: Reporting Quality of Randomized Controlled Trials in Antineutrophil Cytoplasmic Autoantibody–Associated Vasculitis
Source: Kidney Int Rep. 2025 Jul 16;10(10):3484–93. doi: 10.1016/j.ekir.2025.07.012 (PMC12545635; doi:10.1016/j.ekir.2025.07.012)
Supplement: Supplementary File (PDF) — Table S1. Main characteristics of all included trials. List of all randomized controlled trials included in the analysis. CONSORT 2010 checklist. CONSORT-Abstracts checklist. [file mmc1.pdf]

Supplementary Material

Reporting quality of randomized controlled trials in ANCA-associated vasculitis

Vanja Ivković[1], Annette Bruchfeld[1,2], Lauren Floyd[3], Sarah Soyeon Oh[4], Peong Gang Park[5], Jae Il Shin[6,*], Andreas Kronbichler[1,7,*]

[1]Department of Health, Medicine and Caring Sciences, Linköping University, Linköping, Sweden.

[2]Department of Renal Medicine, Karolinska University Hospital and CLINTEC Karolinska Institutet, Stockholm, Sweden

[3]Department of Renal Medicine, Royal Preston Hospital, Lancashire Teaching Hospitals NHS Foundation Trust, UK

[4]Institute of Global Engagement & Empowerment, Yonsei University, Seoul, Republic of Korea

[5]Department of Pediatrics, Ajou University School of Medicine, Suwon, Republic of Korea

[6]Department of Pediatrics, Yonsei University College of Medicine, Seoul 03722, Republic of Korea; Division of Pediatric Nephrology, Severance Children's Hospital, Seoul 03722, Republic of Korea; Institute of Kidney Disease Research, Yonsei University College of Medicine, Seoul 03722, Republic of Korea

[7]Department of Internal Medicine IV, Nephrology and Hypertension, Medical University Innsbruck, Innsbruck, Austria

Supplementary table 1. Main characteristics of all included trials.

First Author, year	*Journal*	*Single- or multicenter*	*Primary outcome*	*Interven-tion type*	*Active arm*	*Comparator arm*	*Total patients*	*CONSORT 2010 score*	*CONSORT 2010 adherence (%)*	*CONSORT-A adherence score*	*CONSORT-A adherence (%)*
Wechsler, 2024	N Engl J Med	multicenter	Remission at weeks 36 and 48	Drug	benralizumab	meprolizumab	140	27	77.1%	16	94.1%
Zonozi, 2024	Ann Rheum Dis	single-center	Relapse by 36 months	Drug	rituximab (monitoring B-cell repopulation)	rituximab (monitoring ANCA rise)	115	29	85.3%	12	70.6%
Garbe, 2023	Scand J Rheumatol	single-center	Mean difference	Other	seminar immediately	seminar after waiting several months	50	21	72.4%	8	50.0%
Smith, 2023	Ann Rheum Dis	multicenter	Preventing relapse	Drug	rituximab	azathioprine	170	27	79.4%	15	88.2%
Arimura, 2023	Mod Rheumatol	multicenter	MMT sum score difference	Drug	intravenous immunoglobulin	placebo	37	32	94.1%	13	76.5%
Mase, 2022	Clin Exp Nephrol	multicenter	Relapse of MPO-AAV at 1 year	Drug	mizoribine	none (standard of care)	53	20	60.6%	11	64.7%
Gopaluni, 2022	Arthritis Res Ther	single-center	Complete or partial response at 6 months	Drug	alemtuzumab (high-dose)	alemtuzumab (low-dose)	23	25	73.5%	15	88.2%
Furuta, 2021	JAMA	multicenter	Remission at 6 months	Drug	rituximab + reduced-dose glucocorticoid	rituximab + high-dose glucocorticoid	134	28	84.8%	16	94.1%
Jayne, 2021	N Engl J Med	multicenter	Remission at 24 weeks	Drug	avacopan	prednisone	330	30	85.7%	16	94.1%
Harper, 2021	Rheumatology (Oxford)	single-center	Recruitment, adherence and drop-out with predefined stop/go criteria	Other	physical activity sessions and telephone calls	standard of care	43	24	75.0%	13	76.5%
Merkel, 2020	ACR Open Rheumatol	multicenter	BVAS reduction by at least 50% at day 85*	Drug	avacopan (2 arms)	placebo	42	29	80.6%	14	82.4%

Charles, 2020	Ann Intern Med	multicenter	Relapse-free survival at month 28	Drug	rituximab	placebo	97	30	83.3%	14	82.4%
Walsh, 2020	N Engl J Med	multicenter	Death from any cause or ESKD	Other	PLEX	no PLEX	704	30	88.2%	15	88.2%
Tuin, 2019	Clin J Am Soc Nephrol	multicenter	Remission at 6 months	Drug	MMF	cyclophospha mide	84	30	83.3%	10	58.8%
Jayne, 2019	Arthritis Rheumatol	multicenter	Time to BVAS≥6, presence of ≥1 major BVAS item, or receipt of prohibited medications for any reason, resulting in treatment failure	Drug	belimumab	placebo	105	32	86.5%	14	82.4%
Jones, 2019	Ann Rheum Dis	multicenter	Remission at 6 months	Drug	MMF	cyclophospha mide	140	29	85.3%	14	82.4%
Chanouzas, 2019	J Infect Dis	single-center	Proportion of patients with CMV reactivation in 6 months	Drug	valacyclovir	standard of care	38	29	87.9%	8	47.1%
Charles, 2018	Ann Rheum Dis	multicenter	Relapse at 28 months	Drug	individually-tailored dosing rituximab	fixed-schedule dosing rituximab	162	30	88.2%	10	58.8%
Maritati, 2017	PLoS One	single-center	Relapse at 12 months	Drug	methotrexate	cyclophospha mide	71	28	87.5%	13	76.5%
Puéchal, 2017	Arthritis Rheumatol	multicenter	Combined rate of remission-induction failures and minor or major relapses at 24 months	Drug	azathioprine	placebo	95	29	82.9%	14	82.4%
Karras, 2017	Ann Rheum Dis	multicenter	Relapse at 48 months	Drug	azathioprine and prednisolone (prolonged treatment)	azathioprine and prednisolone (shorter treatment)	110	26	78.8%	11	64.7%

Wechsler, 2017	N Engl J Med	multicenter	Remission weeks accrued	Drug	mepolizumab	placebo	136	31	86.1%	16	94.1%
Jayne, 2017	J Am Soc Nephrol	multicenter	Proportion of patients with a treatment response at week 12 defined as a BVAS decrease from baseline of at least 50% plus no worsening in any body system		avacopan	placebo	67	31	88.6%	12	70.6%
Sanders, 2016	Nephrol Dial Transplant	multicenter	Time from remission to first relapse, at 4 years post-diagnosis	Drug	azathioprine (extended dosing)	azathioprine (standard dosing)	45	26	76.5%	12	70.6%
Pagnoux, 2015	Arthritis Rheumatol	multicenter	Occurrence of at least 1 SAE during 3 years of follow-up	Drug	azathioprine-containing regimen	standard-of-care	104	28	82.4%	13	76.5%
Jeffs, 2015	Nephrology (Carlton)	multicenter	Reactivation of disease at 28 days	Drug	Influenza vaccine	None (no vaccine)	98	29	90.6%	8	47.1%
Koike, 2015	J Neurol	multicenter	Amount of change in MMT sum score at 2 weeks	Drug	intravenous immunoglobulin	placebo	37	26	74.3%	9	52.9%
Guillevin, 2014	N Engl J Med	multicenter	Relapse at 28 months	Drug	rituximab	azathioprine	115	28	84.8%	12	70.6%
de Menthon, 2011	Clin Exp Rheumatol	multicenter	Partial or complete remission at 12 months	Drug	infliximab	rituximab	17	20	60.6%	11	68.8%

Han, 2011	Am J Nephrol	single-center	Remission rate at 6 months	Drug	MMF	cyclophosphamide	41	22	68.8%	10	58.8%
Szpirt, 2011	Nephrol Dial Transplant	single-center	ESKD or renal progression at 1 month	Other	PLEX	standard therapy	32	25	78.1%	7	41.2%

*Secondary outcome which was used for analyses. Abbreviations: ANCA=antineutrophil cytoplasmic antibody; BVAS=Birmingham Vasculitis Activity Score; CMV=cytomegalovirus; CrCl=creatinine clearance; ESKD=end-stage kidney disease; MMF=mycophenolate mofetil; MMT=manual muscle test; MPO=myeloperoxidase; PLEX=plasma exchange.

Supplementary file 1. List of all randomized controlled trials included in the analysis.

List of randomized controlled trials included in the review

1. Wechsler ME, Nair P, Terrier B, Walz B, Bourdin A, Jayne DRW, Jackson DJ, Roufosse F, Börjesson Sjö L, Fan Y, Jison M, McCrae C, Necander S, Shavit A, Walton C, Merkel PA; MANDARA Study Group. Benralizumab versus Mepolizumab for Eosinophilic Granulomatosis with Polyangiitis. N Engl J Med. 2024 Mar 7;390(10):911-921. doi: 10.1056/NEJMoa2311155. Epub 2024 Feb 23. PMID: 38393328.

2. Zonozi R, Cortazar FB, Jeyabalan A, Sauvage G, Nithagon P, Huizenga NR, Rosenthal JM, Sipilief A, Cosgrove K, Laliberte KA, Rhee EP, Pendergraft WF 3rd, Niles JL. Maintenance of remission of ANCA vasculitis by rituximab based on B cell repopulation versus serological flare: a randomised trial. Ann Rheum Dis. 2024 Feb 15;83(3):351-359. doi: 10.1136/ard-2023-224489. PMID: 38123922.

3. Arimura Y, Sobue G, Hattori N, Takashima H, Harigai M, Nagata K, Makino H. Intravenous immunoglobulin for chronic residual peripheral neuropathy in microscopic polyangiitis: A multicentre randomised double-blind trial. Mod Rheumatol. 2023 Nov 1;33(6):1125-1136. doi: 10.1093/mr/roac137. PMID: 36346309.

4. Smith RM, Jones RB, Specks U, Bond S, Nodale M, Al-Jayyousi R, Andrews J, Bruchfeld A, Camilleri B, Carette S, Cheung CK, Derebail V, Doulton T, Ferraro A, Forbess L, Fujimoto S, Furuta S, Gewurz-Singer O, Harper L, Ito-Ihara T, Khalidi N, Klocke R, Koening C, Komagata Y, Langford C, Lanyon P, Luqmani R, McAlear C, Moreland LW, Mynard K, Nachman P, Pagnoux C, Peh CA, Pusey C, Ranganathan D, Rhee RL, Spiera R, Sreih AG, Tesar V, Walters G, Wroe C, Jayne D, Merkel PA; RITAZAREM co investigators. Rituximab versus azathioprine for maintenance of remission for patients with ANCA-associated vasculitis and relapsing disease: an international randomised controlled trial. Ann Rheum Dis. 2023 Jul;82(7):937-944. doi: 10.1136/ard-2022-223559. Epub 2023 Mar 23. PMID: 36958796; PMCID: PMC10313987.

5. Garbe N, Schäfer C, Pilz A, Schmidt F, Raberger K, Wienke A, Keyßer G. The impact of a structured one-day seminar on disease-specific knowledge, lifestyle habits and disease impairment in ANCA-associated vasculitis. Results of a randomized, controlled study. Scand J Rheumatol. 2023 Jan;52(1):69-76. doi: 10.1080/03009742.2021.1972534. Epub 2021 Oct 13. PMID: 34643169.

6. Mase K, Saito C, Usui J, Arimura Y, Nitta K, Wada T, Makino H, Muso E, Hirawa N, Kobayashi M, Yumura W, Fujimoto S, Nakagawa N, Ito T, Yuzawa Y, Matsuo S, Yamagata K. The efficacy and safety of mizoribine for maintenance therapy in patients with myeloperoxidase anti-neutrophil cytoplasmic antibody (MPO- ANCA)-associated vasculitis: the usefulness of serum mizoribine monitoring. Clin Exp Nephrol. 2022 Nov;26(11):1092-1099. doi: 10.1007/s10157-022-02253-6. Epub 2022 Jul 30. PMID: 35908130.

7. Gopaluni S, Smith R, Goymer D, Cahill H, Broadhurst E, Wallin E, McClure M, Chaudhry A, Jayne D. Alemtuzumab for refractory primary systemic vasculitis-a randomised controlled dose ranging clinical trial of efficacy and safety (ALEVIATE). Arthritis Res Ther. 2022 Apr 1;24(1):81. doi: 10.1186/s13075-022-02761-6. PMID: 35365179; PMCID: PMC8972754.

8. Harper L, Hewitt CA, Litchfield I, Morgan MD, Chanouzas D, Caulfield HK, Coughlan L, Dean C, Fletcher K, Cramp F, Greenfield S, Ives NJ, Jowett S, Kodabuckus S, Tearne S, Sehmi S, Edwardson C, Dawkins NP, Daley AJ. Management of fatigue with physical activity and behavioural change support in vasculitis: a feasibility study. Rheumatology (Oxford). 2021 Sep 1;60(9):4130-4140. doi: 10.1093/rheumatology/keaa890. PMID: 33369680; PMCID: PMC8410006.

9. Furuta S, Nakagomi D, Kobayashi Y, Hiraguri M, Sugiyama T, Amano K, Umibe T, Kono H, Kurasawa K, Kita Y, Matsumura R, Kaneko Y, Ninagawa K, Hiromura K, Kagami SI, Inaba Y, Hanaoka H, Ikeda K, Nakajima H; LoVAS Collaborators. Effect of Reduced-Dose vs High-Dose Glucocorticoids Added to Rituximab on Remission Induction in ANCA-Associated Vasculitis: A Randomized Clinical Trial. JAMA. 2021 Jun 1;325(21):2178-2187. doi: 10.1001/jama.2021.6615. PMID: 34061144; PMCID: PMC8170547.

10. Jayne DRW, Merkel PA, Schall TJ, Bekker P; ADVOCATE Study Group. Avacopan for the Treatment of ANCA-Associated Vasculitis. N Engl J Med. 2021 Feb 18;384(7):599-609. doi: 10.1056/NEJMoa2023386. Erratum in: N Engl J Med. 2024 Jan 25;390(4):388. doi: 10.1056/NEJMx230010. PMID: 33596356.

11. Merkel PA, Niles J, Jimenez R, Spiera RF, Rovin BH, Bomback A, Pagnoux C, Potarca A, Schall TJ, Bekker P; CLASSIC Investigators. Adjunctive Treatment With Avacopan, an Oral C5a Receptor Inhibitor, in Patients With Antineutrophil Cytoplasmic Antibody-Associated Vasculitis. ACR Open Rheumatol. 2020 Nov;2(11):662-671. doi: 10.1002/acr2.11185. Epub 2020 Oct 31. PMID: 33128347; PMCID: PMC7672305.

12. Charles P, Perrodeau É, Samson M, Bonnotte B, Néel A, Agard C, Huart A, Karras A, Lifermann F, Godmer P, Cohen P, Hanrotel-Saliou C, Martin-Silva N, Pugnet G, Maurier F, Sibilia J, Carron PL, Gobert P, Meaux-Ruault N, Le Gallou T, Vinzio S, Viallard JF, Hachulla E, Vinter C, Puéchal X, Terrier B, Ravaud P, Mouthon L, Guillevin L; French Vasculitis Study Group. Long-Term Rituximab Use to Maintain Remission of Antineutrophil Cytoplasmic Antibody-Associated Vasculitis: A Randomized Trial. Ann Intern Med. 2020 Aug 4;173(3):179-187. doi: 10.7326/M19-3827. Epub 2020 Jun 2. PMID: 32479166.

13. Walsh M, Merkel PA, Peh CA, Szpirt WM, Puéchal X, Fujimoto S, Hawley CM, Khalidi N, Floßmann O, Wald R, Girard LP, Levin A, Gregorini G, Harper L, Clark WF, Pagnoux C, Specks U, Smyth L, Tesar V, Ito-Ihara T, de Zoysa JR, Szczeklik W, Flores-Suárez LF, Carette S, Guillevin L, Pusey CD, Casian AL, Brezina B, Mazzetti A, McAlear CA, Broadhurst E, Reidlinger D, Mehta S, Ives N, Jayne DRW; PEXIVAS Investigators. Plasma Exchange and Glucocorticoids in Severe ANCA- Associated Vasculitis. N Engl J Med. 2020 Feb 13;382(7):622-631. doi: 10.1056/NEJMoa1803537. PMID: 32053298; PMCID: PMC7325726.

14. Tuin J, Stassen PM, Bogdan DI, Broekroelofs J, van Paassen P, Cohen Tervaert JW, Sanders JS, Stegeman CA. Mycophenolate Mofetil Versus Cyclophosphamide for the Induction of Remission in Nonlife-Threatening Relapses of Antineutrophil Cytoplasmic Antibody-Associated Vasculitis: Randomized, Controlled Trial. Clin J Am Soc Nephrol. 2019 Jul 5;14(7):1021-1028. doi: 10.2215/CJN.11801018. Epub 2019 Jun 28. PMID: 31253599; PMCID: PMC6625631.

15. Jayne D, Blockmans D, Luqmani R, Moiseev S, Ji B, Green Y, Hall L, Roth D, Henderson RB, Merkel PA; BREVAS Study Collaborators. Efficacy and Safety of Belimumab and Azathioprine for Maintenance of Remission in Antineutrophil Cytoplasmic Antibody-Associated Vasculitis: A Randomized Controlled Study. Arthritis Rheumatol. 2019 Jun;71(6):952-963. doi: 10.1002/art.40802. Epub 2019 Apr 16. PMID: 30666823; PMCID: PMC6593987.

16. Jones RB, Hiemstra TF, Ballarin J, Blockmans DE, Brogan P, Bruchfeld A, Cid MC, Dahlsveen K, de Zoysa J, Espigol-Frigolé G, Lanyon P, Peh CA, Tesar V, Vaglio A, Walsh M, Walsh D, Walters G, Harper L, Jayne D; European Vasculitis Study Group (EUVAS). Mycophenolate mofetil versus cyclophosphamide for remission induction in ANCA-associated vasculitis: a randomised, non-inferiority trial. Ann Rheum Dis. 2019 Mar;78(3):399-405. doi: 10.1136/annrheumdis-2018-214245. Epub 2019 Jan 5. PMID: 30612116.

17. Chanouzas D, Sagmeister M, Faustini S, Nightingale P, Richter A, Ferro CJ, Morgan MD, Moss P, Harper L. Subclinical Reactivation of Cytomegalovirus Drives CD4+CD28null T-Cell Expansion and Impaired Immune Response to Pneumococcal Vaccination in Antineutrophil Cytoplasmic Antibody-Associated Vasculitis. J Infect Dis. 2019 Jan 7;219(2):234-244. doi: 10.1093/infdis/jiy493. PMID: 30102389; PMCID: PMC6306020.

18. Charles P, Terrier B, Perrodeau É, Cohen P, Faguer S, Huart A, Hamidou M, Agard C, Bonnotte B, Samson M, Karras A, Jourde-Chiche N, Lifermann F, Gobert P, Hanrotel-Saliou C, Godmer P, Martin-Silva N, Pugnet G, Matignon M, Aumaitre O, Viallard JF, Maurier F, Meaux-Ruault N, Rivière S, Sibilia J, Puéchal X, Ravaud P, Mouthon L, Guillevin L; French Vasculitis Study Group. Comparison of individually tailored versus fixed-schedule rituximab regimen to maintain ANCA- associated vasculitis remission:

results of a multicentre, randomized controlled, phase III trial (MAINRITSAN2). Ann Rheum Dis. 2018 Aug;77(8):1143-1149. doi: 10.1136/annrheumdis-2017-212878. Epub 2018 Apr 25. Erratum in: Ann Rheum Dis. 2019 Sep;78(9):e101. doi: 10.1136/annrheumdis-2017-212878corr1. PMID: 29695500.

19. Puéchal X, Pagnoux C, Baron G, Quémeneur T, Néel A, Agard C, Lifermann F, Liozon E, Ruivard M, Godmer P, Limal N, Mékinian A, Papo T, Ruppert AM, Bourgarit A, Bienvenu B, Geffray L, Saraux JL, Diot E, Crestani B, Delbrel X, Sailler L, Cohen P, Le Guern V, Terrier B, Groh M, Le Jeunne C, Mouthon L, Ravaud P, Guillevin L; French Vasculitis Study Group. Adding Azathioprine to Remission-Induction Glucocorticoids for Eosinophilic Granulomatosis With Polyangiitis (Churg-Strauss), Microscopic Polyangiitis, or Polyarteritis Nodosa Without Poor Prognosis Factors: A Randomized, Controlled Trial. Arthritis Rheumatol. 2017 Nov;69(11):2175-2186. doi: 10.1002/art.40205. Epub 2017 Oct 15. PMID: 28678392.

20. Maritati F, Alberici F, Oliva E, Urban ML, Palmisano A, Santarsia F, Andrulli S, Pavone L, Pesci A, Grasselli C, Santi R, Tumiati B, Manenti L, Buzio C, Vaglio A. Methotrexate versus cyclophosphamide for remission maintenance in ANCA-associated vasculitis: A randomised trial. PLoS One. 2017 Oct 10;12(10):e0185880. doi: 10.1371/journal.pone.0185880. PMID: 29016646; PMCID: PMC5634660.

21. Karras A, Pagnoux C, Haubitz M, Groot K, Puechal X, Tervaert JWC, Segelmark M, Guillevin L, Jayne D; European Vasculitis Society. Randomised controlled trial of prolonged treatment in the remission phase of ANCA-associated vasculitis. Ann Rheum Dis. 2017 Oct;76(10):1662-1668. doi: 10.1136/annrheumdis-2017-211123. Epub 2017 May 25. PMID: 28546260.

22. Jayne DRW, Bruchfeld AN, Harper L, Schaier M, Venning MC, Hamilton P, Burst V, Grundmann F, Jadoul M, Szombati I, Tesař V, Segelmark M, Potarca A, Schall TJ, Bekker P; CLEAR Study Group. Randomized Trial of C5a Receptor Inhibitor Avacopan in ANCA-Associated Vasculitis. J Am Soc Nephrol. 2017 Sep;28(9):2756-2767. doi: 10.1681/ASN.2016111179. Epub 2017 Apr 11. PMID: 28400446; PMCID: PMC5576933.

23. Wechsler ME, Akuthota P, Jayne D, Khoury P, Klion A, Langford CA, Merkel PA, Moosig F, Specks U, Cid MC, Luqmani R, Brown J, Mallett S, Philipson R, Yancey SW, Steinfeld J, Weller PF, Gleich GJ; EGPA Mepolizumab Study Team. Mepolizumab or Placebo for Eosinophilic Granulomatosis with Polyangiitis. N Engl J Med. 2017 May 18;376(20):1921-1932. doi: 10.1056/NEJMoa1702079. PMID: 28514601; PMCID: PMC5548295.

24. Sanders JS, de Joode AA, DeSevaux RG, Broekroelofs J, Voskuyl AE, van Paassen P, Kallenberg CG, Tervaert JW, Stegeman CA. Extended versus standard azathioprine maintenance therapy in newly diagnosed proteinase-3 anti-neutrophil cytoplasmic antibody-

associated vasculitis patients who remain cytoplasmic anti- neutrophil cytoplasmic antibody-positive after induction of remission: a randomized clinical trial. Nephrol Dial Transplant. 2016 Sep;31(9):1453-9. doi: 10.1093/ndt/gfw211. Epub 2016 May 30. PMID: 27242368.

25. Jeffs LS, Peh CA, Jose MD, Lange K, Hurtado PR. Randomized trial investigating the safety and efficacy of influenza vaccination in patients with antineutrophil cytoplasmic antibody-associated vasculitis. Nephrology (Carlton). 2015 May;20(5):343-51. doi: 10.1111/nep.12416. PMID: 25656094.

26. Pagnoux C, Quéméneur T, Ninet J, Diot E, Kyndt X, de Wazières B, Reny JL, Puéchal X, le Berruyer PY, Lidove O, Vanhille P, Godmer P, Fain O, Blockmans D, Bienvenu B, Rollot F, Aït el Ghaz-Poignant S, Mahr A, Cohen P, Mouthon L,Perrodeau E, Ravaud P, Guillevin L; French Vasculitis Study Group. Treatment of systemic necrotizing vasculitides in patients aged sixty-five years or older: results of a multicenter, open-label, randomized controlled trial of corticosteroid and cyclophosphamide-based induction therapy. Arthritis Rheumatol. 2015 Apr;67(4):1117-27. doi: 10.1002/art.39011. PMID: 25693055.

27. Koike H, Akiyama K, Saito T, Sobue G; Research Group for IVIg for EGPA/CSS in Japan. Intravenous immunoglobulin for chronic residual peripheral neuropathy in eosinophilic granulomatosis with polyangiitis (Churg-Strauss syndrome): a multicenter, double-blind trial. J Neurol. 2015 Mar;262(3):752-9. doi: 10.1007/s00415-014-7618-y. Epub 2015 Jan 11. PMID: 25577176; PMCID: PMC4363522.

28. Guillevin L, Pagnoux C, Karras A, Khouatra C, Aumaître O, Cohen P, Maurier F, Decaux O, Ninet J, Gobert P, Quémeneur T, Blanchard-Delaunay C, Godmer P, Puéchal X, Carron PL, Hatron PY, Limal N, Hamidou M, Ducret M, Daugas E, Papo T, Bonnotte B, Mahr A, Ravaud P, Mouthon L; French Vasculitis Study Group. Rituximab versus azathioprine for maintenance in ANCA-associated vasculitis. N Engl J Med. 2014 Nov 6;371(19):1771-80. doi: 10.1056/NEJMoa1404231. PMID: 25372085.

29. de Menthon M, Cohen P, Pagnoux C, Buchler M, Sibilia J, Détrée F, Gayraud M, Khellaf M, Penalba C, Legallicier B, Mouthon L, Guillevin L. Infliximab or rituximab for refractory Wegener's granulomatosis: long-term follow up. A prospective randomised multicentre study on 17 patients. Clin Exp Rheumatol. 2011 Jan-Feb;29(1 Suppl 64):S63-71. Epub 2011 May 11. PMID: 21586199.

30. Han F, Liu G, Zhang X, Li X, He Q, He X, Li Q, Wang S, Wang H, Chen J. Effects of mycophenolate mofetil combined with corticosteroids for induction therapy of microscopic polyangiitis. Am J Nephrol. 2011;33(2):185-92. doi: 10.1159/000324364. Epub 2011 Feb 11. PMID: 21311184.

31. Szpirt WM, Heaf JG, Petersen J. Plasma exchange for induction and cyclosporine A for maintenance of remission in Wegener's granulomatosis—a clinical randomized controlled trial. Nephrol Dial Transplant. 2011 Jan;26(1):206-13. doi: 10.1093/ndt/gfq360. Epub 2010 Jun 24. PMID: 20577017.

Supplementary file 2. CONsolidated Standards of Reporting Trials (CONSORT) 2010 checklist of information to include when reporting a randomized trial.

CONSORT 2010 checklist of information to include when reporting a randomised trial*

Section/Topic	Item No	Checklist item	Reported on page No
**Title and abstract**			
	1a	Identification as a randomised trial in the title	
	1b	Structured summary of trial design, methods, results, and conclusions (for specific guidance see CONSORT for abstracts)	
**Introduction**			
Background and objectives	2a	Scientific background and explanation of rationale	
	2b	Specific objectives or hypotheses	
**Methods**			
Trial design	3a	Description of trial design (such as parallel, factorial) including allocation ratio	
	3b	Important changes to methods after trial commencement (such as eligibility criteria), with reasons	
Participants	4a	Eligibility criteria for participants	
	4b	Settings and locations where the data were collected	
Interventions	5	The interventions for each group with sufficient details to allow replication, including how and when they were actually administered	
Outcomes	6a	Completely defined pre-specified primary and secondary outcome measures, including how and when they were assessed	
	6b	Any changes to trial outcomes after the trial commenced, with reasons	
Sample size	7a	How sample size was determined	
	7b	When applicable, explanation of any interim analyses and stopping guidelines	
Randomisation:			
Sequence generation	8a	Method used to generate the random allocation sequence	
	8b	Type of randomisation; details of any restriction (such as blocking and block size)	
Allocation concealment mechanism	9	Mechanism used to implement the random allocation sequence (such as sequentially numbered containers), describing any steps taken to conceal the sequence until interventions were assigned	
Implementation	10	Who generated the random allocation sequence, who enrolled participants, and who assigned participants to interventions	
Blinding	11a	If done, who was blinded after assignment to interventions (for example, participants, care providers, those	

		assessing outcomes) and how	
	11b	If relevant, description of the similarity of interventions	
Statistical methods	12a	Statistical methods used to compare groups for primary and secondary outcomes	
	12b	Methods for additional analyses, such as subgroup analyses and adjusted analyses	
**Results**			
Participant flow (a diagram is strongly recommended)	13a	For each group, the numbers of participants who were randomly assigned, received intended treatment, and were analysed for the primary outcome	
	13b	For each group, losses and exclusions after randomisation, together with reasons	
Recruitment	14a	Dates defining the periods of recruitment and follow-up	
	14b	Why the trial ended or was stopped	
Baseline data	15	A table showing baseline demographic and clinical characteristics for each group	
Numbers analysed	16	For each group, number of participants (denominator) included in each analysis and whether the analysis was by original assigned groups	
Outcomes and estimation	17a	For each primary and secondary outcome, results for each group, and the estimated effect size and its precision (such as 95% confidence interval)	
	17b	For binary outcomes, presentation of both absolute and relative effect sizes is recommended	
Ancillary analyses	18	Results of any other analyses performed, including subgroup analyses and adjusted analyses, distinguishing pre-specified from exploratory	
Harms	19	All important harms or unintended effects in each group (for specific guidance see CONSORT for harms)	
**Discussion**			
Limitations	20	Trial limitations, addressing sources of potential bias, imprecision, and, if relevant, multiplicity of analyses	
Generalisability	21	Generalisability (external validity, applicability) of the trial findings	
Interpretation	22	Interpretation consistent with results, balancing benefits and harms, and considering other relevant evidence	
**Other information**			
Registration	23	Registration number and name of trial registry	
Protocol	24	Where the full trial protocol can be accessed, if available	
Funding	25	Sources of funding and other support (such as supply of drugs), role of funders	

*We strongly recommend reading this statement in conjunction with the CONSORT 2010 Explanation and Elaboration for important clarifications on all the items. If relevant, we also recommend reading CONSORT extensions for cluster randomised trials, non-inferiority and equivalence trials, non-pharmacological treatments, herbal interventions, and pragmatic trials. Additional extensions are forthcoming: for those and for up to date references relevant to this checklist, see www.consort-statement.org.

Supplementary file 3. CONsolidated Standards of Reporting Trials (CONSORT) for reporting randomized trials in journal and conference abstracts.

Checklist Item	Item Number	Description
**Title**	1*	Identification of the study as a trial (inc. phase of the trial)
**Authors**	2	Contact details for the corresponding author
**Trial Design**	3	Description of the trial design (e.g. parallel, cluster, non-inferiority)
Participants	4	Eligibility criteria for participants and the settings where the data were collected
Interventions	5	Interventions intended for each group
Objective	6	Specific objective or hypothesis
Outcome	7	Clearly defined primary outcome for this report
Randomisation*	8	How participants were allocated to interventions
Blinding (Masking)*	9	Whether or not participants, care givers, and those assessing the outcomes were blinded to group assignment
Numbers Randomised*	10	Number of participants in each group
Recruitment	11	Trial Status
Numbers Analysed	12	Number of participants analysed in each group
Outcome	13	For the primary outcome, a result for each group and the estimated effect size and its precision
Harms	14	Important adverse events or side effects
**Conclusions**	15	General interpretation of the results
**Trial Registration**	16	Registration number and name of trial register
**Funding**	17	Source of funding

Supplementary file 4. PRISMA checklist.

PRISMA 2020 Checklist

Section and Topic	Item #	Checklist item	Location where item is reported
**TITLE**			
Title	1	Identify the report as a systematic review.	1
**ABSTRACT**			
Abstract	2	See the PRISMA 2020 for Abstracts checklist.	3
**INTRODUCTION**			
Rationale	3	Describe the rationale for the review in the context of existing knowledge.	5
Objectives	4	Provide an explicit statement of the objective(s) or question(s) the review addresses.	5
**METHODS**			
Eligibility criteria	5	Specify the inclusion and exclusion criteria for the review and how studies were grouped for the syntheses.	6
Information sources	6	Specify all databases, registers, websites, organisations, reference lists and other sources searched or consulted to identify studies. Specify the date when each source was last searched or consulted.	6
Search strategy	7	Present the full search strategies for all databases, registers and websites, including any filters and limits used.	6
Selection process	8	Specify the methods used to decide whether a study met the inclusion criteria of the review, including how many reviewers screened each record and each report retrieved, whether they worked independently, and if applicable, details of automation tools used in the process.	6-7
Data collection process	9	Specify the methods used to collect data from reports, including how many reviewers collected data from each report, whether they worked independently, any processes for obtaining or confirming data from study investigators, and if applicable, details of automation tools used in the process.	7
Data items	10a	List and define all outcomes for which data were sought. Specify whether all results that were compatible with each outcome domain in each study were sought (e.g. for all measures, time points, analyses), and if not, the methods used to decide which results to collect.	7
	10b	List and define all other variables for which data were sought (e.g. participant and intervention characteristics, funding sources). Describe any assumptions made about any missing or unclear information.	7
Study risk of bias assessment	11	Specify the methods used to assess risk of bias in the included studies, including details of the tool(s) used, how many reviewers assessed each study and whether they worked independently, and if applicable, details of automation tools used in the process.	NA
Effect measures	12	Specify for each outcome the effect measure(s) (e.g. risk ratio, mean difference) used in the synthesis or presentation of results.	8
Synthesis methods	13a	Describe the processes used to decide which studies were eligible for each synthesis (e.g. tabulating the study intervention characteristics and comparing against the planned groups for each synthesis (item #5)).	7-8
	13b	Describe any methods required to prepare the data for presentation or synthesis, such as handling of missing summary statistics, or data conversions.	7-8
	13c	Describe any methods used to tabulate or visually display results of individual studies and syntheses.	7-8
	13d	Describe any methods used to synthesize results and provide a rationale for the choice(s). If meta-analysis was performed, describe the model(s), method(s) to identify the presence and extent of statistical heterogeneity, and software package(s) used.	7-8
	13e	Describe any methods used to explore possible causes of heterogeneity among study results (e.g. subgroup analysis, meta-regression).	NA
	13f	Describe any sensitivity analyses conducted to assess robustness of the synthesized results.	NA
Reporting bias assessment	14	Describe any methods used to assess risk of bias due to missing results in a synthesis (arising from reporting biases).	NA
Certainty assessment	15	Describe any methods used to assess certainty (or confidence) in the body of evidence for an outcome.	NA

PRISMA 2020 Checklist

Section and Topic	Item #	Checklist item	Location where item is reported
**RESULTS**			
Study selection	16a	Describe the results of the search and selection process, from the number of records identified in the search to the number of studies included in the review, ideally using a flow diagram.	Figure 1
	16b	Cite studies that might appear to meet the inclusion criteria, but which were excluded, and explain why they were excluded.	Figure 1
Study characteristics	17	Cite each included study and present its characteristics.	NA
Risk of bias in studies	18	Present assessments of risk of bias for each included study.	NA
Results of individual studies	19	For all outcomes, present, for each study: (a) summary statistics for each group (where appropriate) and (b) an effect estimate and its precision (e.g. confidence/credible interval), ideally using structured tables or plots.	Table 1
Results of syntheses	20a	For each synthesis, briefly summarise the characteristics and risk of bias among contributing studies.	NA
	20b	Present results of all statistical syntheses conducted. If meta-analysis was done, present for each the summary estimate and its precision (e.g. confidence/credible interval) and measures of statistical heterogeneity. If comparing groups, describe the direction of the effect.	7-11
	20c	Present results of all investigations of possible causes of heterogeneity among study results.	NA
	20d	Present results of all sensitivity analyses conducted to assess the robustness of the synthesized results.	NA
Reporting biases	21	Present assessments of risk of bias due to missing results (arising from reporting biases) for each synthesis assessed.	NA
Certainty of evidence	22	Present assessments of certainty (or confidence) in the body of evidence for each outcome assessed.	NA
**DISCUSSION**			
Discussion	23a	Provide a general interpretation of the results in the context of other evidence.	7-11
	23b	Discuss any limitations of the evidence included in the review.	15
	23c	Discuss any limitations of the review processes used.	15
	23d	Discuss implications of the results for practice, policy, and future research.	12-15
**OTHER INFORMATION**			
Registration and protocol	24a	Provide registration information for the review, including register name and registration number, or state that the review was not registered.	8
	24b	Indicate where the review protocol can be accessed, or state that a protocol was not prepared.	8
	24c	Describe and explain any amendments to information provided at registration or in the protocol.	NA
Support	25	Describe sources of financial or non-financial support for the review, and the role of the funders or sponsors in the review.	15
Competing interests	26	Declare any competing interests of review authors.	15
Availability of data, code and other materials	27	Report which of the following are publicly available and where they can be found: template data collection forms; data extracted from included studies; data used for all analyses; analytic code; any other materials used in the review.	NA

From: Page MJ, McKenzie JE, Bossuyt PM, Boutron I, Hoffmann TC, Mulrow CD, et al. The PRISMA 2020 statement: an updated guideline for reporting systematic reviews. BMJ 2021;372:n71. doi: 10.1136/bmj.n71.
This work is licensed under CC BY 4.0. To view a copy of this license, visit https://creativecommons.org/licenses/by/4.0/
